# The Study of the Degree of Crystallinity, Electrical Equivalent Circuit, and Dielectric Properties of Polyvinyl Alcohol (PVA)-Based Biopolymer Electrolytes

**DOI:** 10.3390/polym12102184

**Published:** 2020-09-24

**Authors:** Shujahadeen B. Aziz, Ayub S. Marf, Elham M. A. Dannoun, Mohamad A. Brza, Ranjdar M. Abdullah

**Affiliations:** 1 Advanced Polymeric Materials Research Lab., Department of Physics, College of Science, University of Sulaimani, Qlyasan Street, Kurdistan Regional Government, Sulaimani 46001, Iraq; ayub.shahab@gmail.com (A.S.M.); ranjdar.abdullah@univsul.edu.iq (R.M.A.); 2 Department of Civil Engineering, College of Engineering, Komar University of Science and Technology, Kurdistan Regional Government, Sulaimani 46001, Iraq; 3 Associate Director of General Science Department, Woman Campus, Prince Sultan University, P.O. Box 66833, Riyadh 11586, Saudi Arabia; elhamdannoun1977@gmail.com; 4 Manufacturing and Materials Engineering Department, Faculty of Engineering, International Islamic University of Malaysia, Kuala Lumpur 50603, Gombak, Malaysia; mohamad.brza@gmail.com

**Keywords:** PVA-chitosan blend, plasticized polymer electrolyte, ammonium iodide, XRD, degree of crystallinity, EIS analysis, dielectric properties.

## Abstract

This report presents a facile and efficient methodology for the fabrication of plasticized polyvinyl alcohol (PVA):chitosan (CS) polymer electrolytes using a solution cast technique. Regarding characterizations of electrical properties and structural behavior, the electrochemical impedance spectroscopy (EIS) and X-ray diffraction (XRD) are used, respectively. Crystalline peaks appear in the XRD pattern of the PVA:CS:NH_4_I while no peaks can be seen in the XRD pattern of plasticized systems. The degree of crystallinity is calculated for all the samples from the deconvoluted area of crystalline and amorphous phases. Considering the EIS measurements, the most conductive plasticized system shows a relatively high conductivity of (1.37 × 10^−4^) S/cm, which is eligible for applications in energy storage devices. The analysis of the EIS spectra reveals a decrease in bulk resistance which indicates an increase in free ion carriers. The electrical equivalent circuit (EEC) model is used in the analysis of EIS plots. Dielectric properties are modified with the addition of glycerol as a plasticizer. It is proved that the addition of glycerol as a plasticizer lowers ion association. It also shows, at the low-frequency region, a large value of a dielectric constant which is correlated with electrode polarization (EP). The distribution of relaxation times is associated with conducting ions.

## 1. Introduction

Solid polymer-based electrolytes as promising electrolytes have increased the awareness of numerous research groups due to the extensive utilization of these advanced materials in electrochemical energy devices; for example, in supercapacitors, dye-sensitized solar cells, fuel cells, and high energy solid-state batteries [1,2]. There are several advantages to solid polymer electrolytes (SPEs) which can be taken into consideration, for instance, extended lifetime, safety, lightweight, mechanical flexibility, high corrosion resistance, and trouble-free processing [3,4,5]. However, the main and effective barrier that makes SPEs hard to utilize on a large scale is the relatively low ionic conductivity at ambient temperatures [6,7]. Therefore, many attempts have focused on enhancing the ionic conductivity of SPE systems. Thus, various salts have been incorporated into different host polymer matrices [8,9,10,11]. Polymer electrolytes are hosts for ion carriers while conductive polymers such as polyaniline (PANI) are hosts for electron carriers [12]. Polymer electrolyte ions are responsible for conduction [9,10,11], while in PANI electrons are sources for conduction [12]. Polymers used for polymer electrolyte preparation can be categorized into natural polymers and synthetic polymers. The natural ones are renewable and can be easily obtained from natural resources, such as proteins, wool, cellulose, and silk. Conversely, the synthetic or man-made polymers can be synthesized from relatively low molecular weight compounds such as monomers, for example, polystyrene and polyethylene. The natural polymers also can be obtained from natural resources just by modifications, for instance, rubber (Hevea), which is known as a polyisoprene in its synthetic form [13].

Poly(vinyl alcohol) (PVA) polymer is one of the fascinating polymers that is characterized by non-toxicity, water solubility, and ability to form a film. Furthermore, many functional groups within the backbone enable PVA to be polar, where it can form hydrogen bonds and facilitate polymer-blend formation [14]. All this means that the hydrophilic character and the high density of reactive chemical functional groups encourage PVA to be able to cross-link with dopant chemical materials [15]. Nowadays, the focus has been devoted to biodegradable and compatible natural polymers, for example, starch, cellulose, chitosan, carrageenan, and agarose. This is due to their environmental sustainability and renewability, and their ease of handling during electrolyte preparations [16,17]. The first and second abundant natural polymers are cellulose and chitin, respectively, the latter being a deacetylated product of chitosan (CS) [18]. CS is extractable from shrimp waste and has received considerable focus in numerous applications [19,20]. It is a polycationic polymer that possesses an amino group and two hydroxyl groups in each repeating unit [21,22,23,24]. Several favorable traits of CS, such as biocompatibility, biodegradability, benign nature, and cheapness cause this material to be under intensive study [25,26]. Based on the functional groups in CS and the high molecular weight polysaccharide, there is a strong network of intermolecular or intramolecular hydrogen bonds [27,28]. The crystalline structure on one side, and the existence of hydrogen bonds on the other side, provides a compromise that it is usually soluble in acids [29,30]. Herein, a promising methodology known as polymer blending has been implemented often to develop new polymeric materials with unique properties that differ from their individual components. This method is usually facile, efficient, and time-saving for synthesizing polymeric materials with desired properties [31,32,33]. Polymer blending is also easy to process, reforming polymers with high flexibility [34]. 

PVA and CS are compatible with blending [35] and they are miscible into each other [36]. The miscibility of PVA and CS blends was established by Lewandowska et al. [37] via dynamic mechanical analysis. It also has been reported that films with high compatibility and miscibility were created when blending PVA with CS [38]. The presence of robust hydrogen bonding among the hydroxyl groups in PVA, and the hydroxyl groups in CS via blending, also offer good mechanical properties [35]. The polymer electrolyte conductivity can be increased by blending two polymers as the host material for ionic conduction [39]. Polymer-blend complexes have a possible application as SPEs in electrochemical devices [40]. Alternatively, plasticization of polymer electrolytes provides a relatively high direct current (DC) conductivity and dielectric constant [41,42]. Chai and Isa [43] added glycerol into carboxymethyl cellulose (CMC) via solution cast methodology, producing plasticized polymer. It was proved that the addition of glycerol improved ionic conductivity and the mechanical strength of the electrolyte film significantly. 

To provide insight into the chemical and physical states of the polymer understudy, dielectric properties, dielectric constant, and dielectric loss have to be measured. To improve these properties, the insertion of dopants into polymers is one of the promising methodologies [12,41,42,44]. As relatively high-energy capacity materials, dielectric polymers have been studied extensively and intensively. The decisive properties of dielectric materials are their large dielectric constant, low dielectric loss, and high electric breakdown strength [45]. Previously, it has been proved that polymer electrolytes with a relatively high DC conductivity and dielectric constant are eligible to be used as separators and charge providers in electrical double layer capacitor (EDLC) applications [12,41,42].

Here, structural, electrical equivalent circuits and several electrical parameters are investigated for PVA:CS:NH_4_I:glycerol systems.

## 2. Experimental Method

### 2.1. Materials and Sample Preparation

Chitosan (CS) obtained from crab shells (≥75% deacetylated, average molecular weight 1.1 × 10^5^ g/mol) and PVA (Mw 89,000–98,000, 99+% hydrolyzed) powder materials were purchased from Sigma–Aldrich (St. Louis, MO, USA). Both polymer raw materials and ammonium iodide (NH_4_I) were obtained from Sigma–Aldrich. The CS powder (0.5 g) was dissolved in 50 mL of 1% acetic acid. Subsequently, the solution was stirred using a magnetic stirrer for 4 h at room temperature until complete dissolution of the polymer, when a clear viscose solution was gained. Separately, an accurate amount of 0.5 g of PVA was dissolved in distilled water with continuous stirring. To prepare series blend solutions, various portions of PVA solutions were added to CS solutions. Then, 40 wt.% of NH_4_I was added into the PVA:CS solution, maintaining stirring. Afterward, various quantities of glycerol ranging from 14 to 42 wt.% as an efficient plasticizer were added to PVA:CS:NH_4_I electrolytes to prepare the plasticized blend electrolytes. The stirring of the mixtures was carried out continuously until homogeneous solutions were achieved. After casting, the coding process was performed in different Petri dishes where the polymer-blend electrolyte samples were labeled as PVCSGP0, PVCSGP1, PVCSGP2, and PVCSGP3 for PVA:CS:NH_4_I incorporated with 0 wt.%, 14 wt.%, 28 wt.%, and 42 wt.% of glycerol, respectively. The solutions were left to dry gradually at room temperature to form films. Ultimately, to ensure the dryness of the films, it was imperative to put them into a desiccator, producing solvent-free films.

### 2.2. Characterization Techniques

The acquisition of X-ray diffraction (XRD) was performed using an X-ray diffractometer (Malvern Panalytical Ltd., Malvern, UK) by applying 40 kV and 40 mA at room temperature. To investigate the electrical properties of the samples, a 3532-50 LCR HiTESTER (Hioki, Nagano, Japan) was used at room temperature. The preferred frequency choice of 0.05–5000 kHz was applied. The electrolyte films were cut into semicircles and sandwiched between two stainless steel (SS) electrodes. 

## 3. Results and Discussion

### 3.1. X-Ray Diffraction (XRD) 

**Figure 1:**
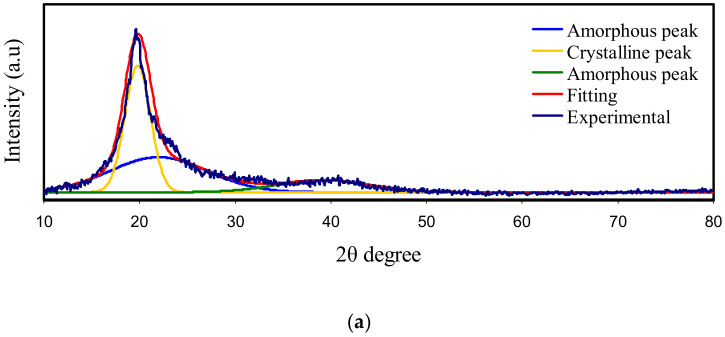

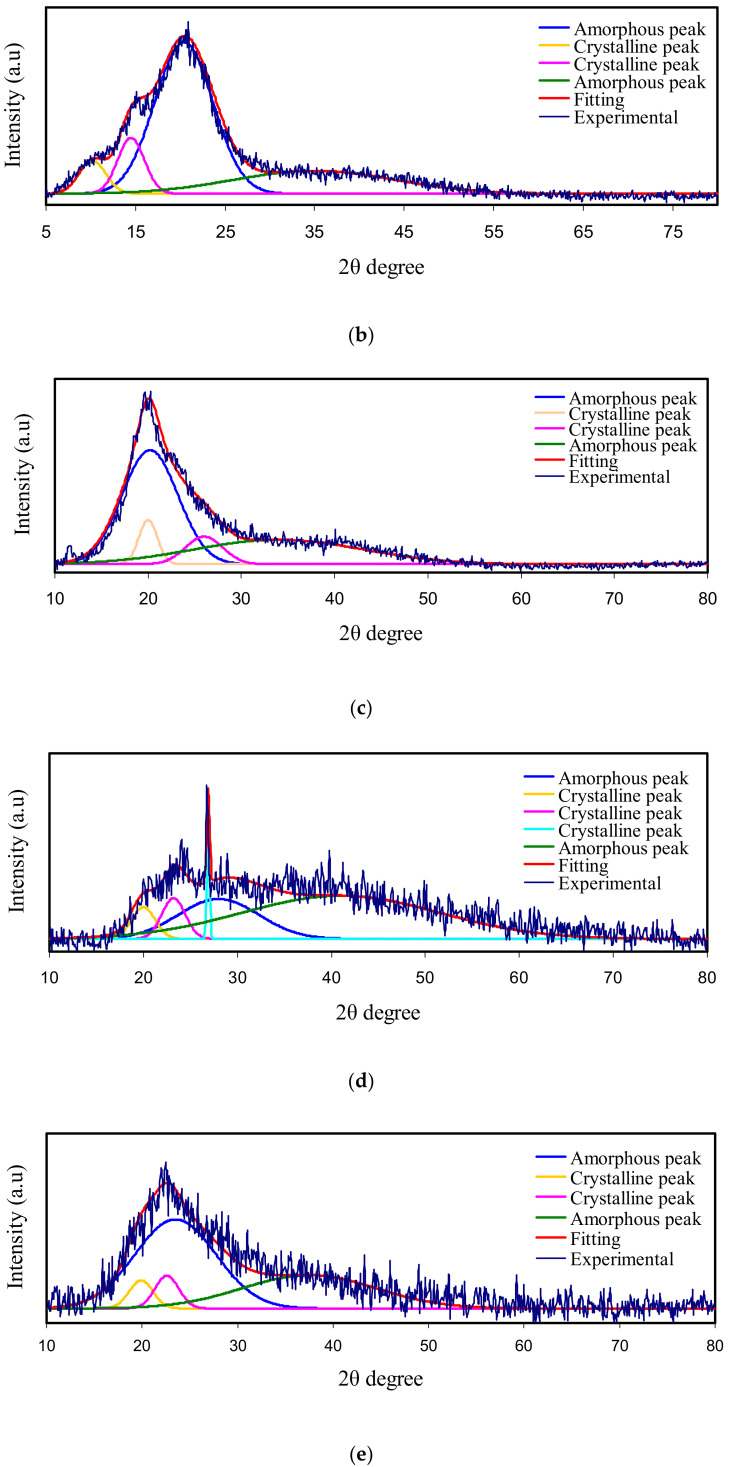

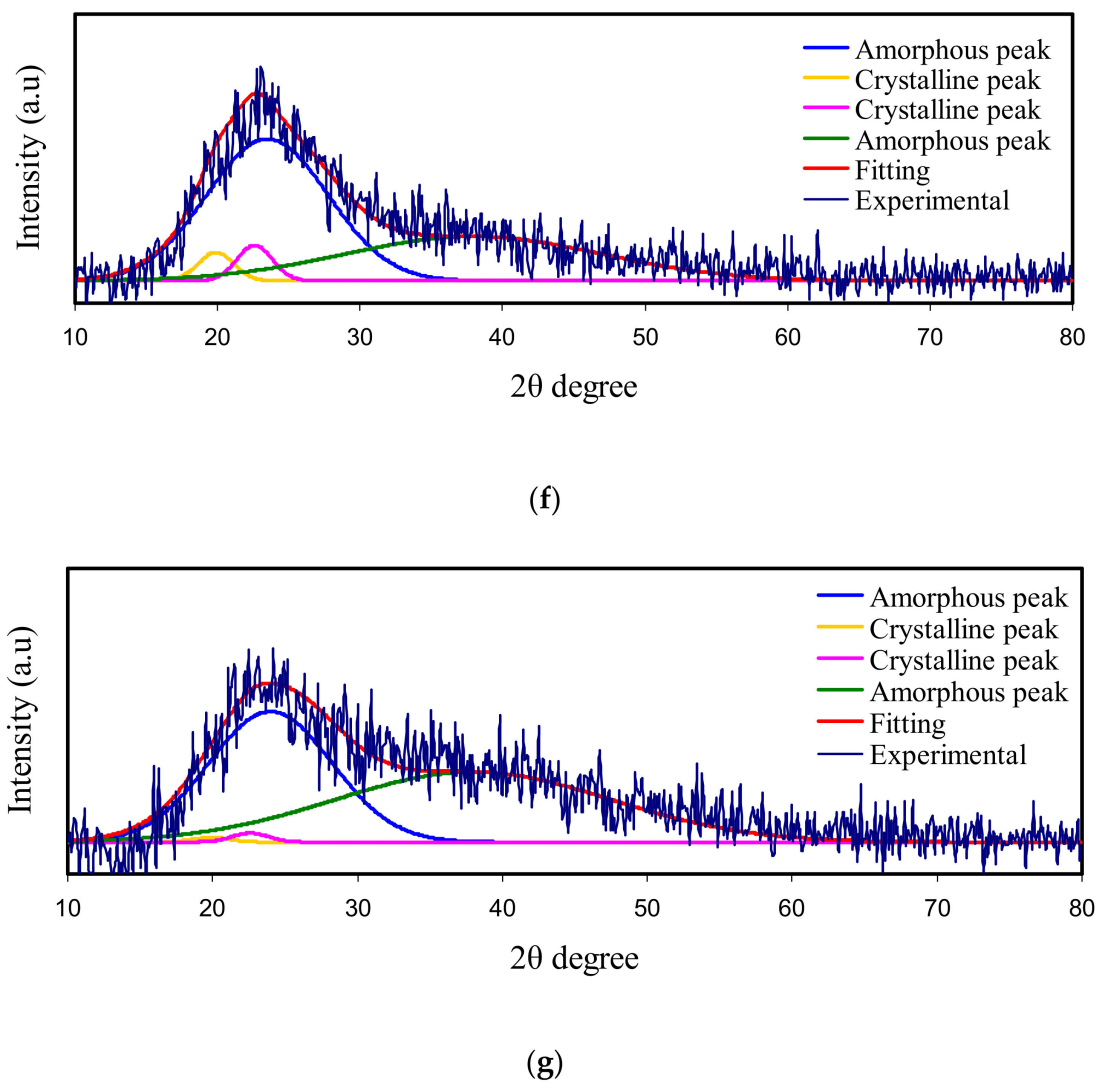
XRD spectra for (**a**) pure PVA, (**b**) pure CS, (**c**) PVA:CS-blend (**d**) PVSCGP0, (**e**) PVSCGP1, (**f**) PVSCGP2, and (**g**) PVSCGP3 electrolyte films.

To examine the influence of glycerol as a plasticizer on the crystalline structure of PVA:CS:NH_4_I, XRD was acquired and analyzed. Early studies showed that the two distinct crystalline peaks at 2θ = 15.1° and 20.9° are a feature of a pure CS sample film. These crystalline peaks at 15.1° and 20.9° correspond to the reflection planes of (110) and (220), respectively [46,47]. The preservation of the rigid crystalline structure of CS is chiefly endorsed to the intramolecular and intermolecular hydrogen bonds, signifying an average intermolecular distance of the crystalline parts of CS [24,48]. The wide peak at 2θ extended from 35 to 55° is the mark of the amorphous phase of CS [49,50,51]. The XRD pattern of pure CS and PVA films are shown in Figure 1a,b. A featured peak around 2θ = 18° proves the semi-crystalline manners of pure PVA [15]. The attachment of PVA with OH groups along the major chain is adequate to have a strong intermolecular and intramolecular hydrogen bonding. Notably, a large peak center at 2θ = 40.7° refers to amorphous phases in the PVA structure. This study showed, as the 50 wt.% of PVA was blended with 50 wt.% of CS, the intensity of the diffraction peaks decreased and broadened (see Figure 1c). This is caused by hydrogen bonding disruption owing to the amorphous structure dominance in the blend system. Therefore, polymer blending could be considered an efficient methodology to reduce the PVA crystalline segment. It is remarkable to see that the degree of crystallinity (*X_C_*) is decreased upon the insertion of CS content (see Table 1). The *X_C_* of CS in this study is quite close to the previous report [52].

Figure 1d shows the XRD pattern for a PVA:CS:NH_4_I electrolyte system. It is seen that the intensity of the peak at 2θ = 18.6° is noticeably decreased, indicating the interaction between the polymer-blend and NH_4_I. However, it is determined that the new strong and high-intensity peak that appears at 2θ = 26.9° is related to the NH_4_I salt, which may be due to the incomplete dissociation of the NH_4_I in the polymer electrolyte film. The XRD pattern of PVA:CS:NH_4_I electrolytes incorporated with a series of quantities of glycerol is shown in Figure 1e–g. It is seen, as the quantity of glycerol is increased up to 42 wt.% into the electrolyte system, the intensity of the XRD peaks is considerably decreased. This indicates decreasing of the *X_C_* of the electrolyte system and the amorphous phase dominates. Moreover, the effect of glycerol as a plasticizer on PVA:CS:NH_4_I, is evidenced from the disruption of hydrogen bonding between the amino groups and the hydroxyl groups in the PVA polymer matrix [53]. Consequently, one can say that plasticizer has a substantial influence on the crystalline phases of PVA solid polymer electrolytes.

To determine the *X_C_*, it is essential to deconvolute the XRD spectra of the samples to find the area of the amorphous and crystalline peaks [47]. The *X_C_* was calculated using Equation (1) [54]:

(1) $$X_{C}=\frac{A_{C}}{A_{T}}\times 100\%$$

where *A_C_* and *A_T_* are the areas of crystalline peaks and the total area of amorphous and crystalline peaks, respectively. It is significant to view that the *X_C_* is reduced upon the inclusion of extra glycerol content (see Table 1). The deconvoluted XRD spectra of plasticized PVA:CS electrolyte is shown in Figure 1a–g. It is fascinating to observe that the intensity of the XRD peaks is considerably decreased. The amorphous structure increment might be associated with the crystalline phase disruption in the polymer [55]. Compared to the pure films, regarding the PVA:CS blend and the un-plasticized system, the *X_C_* in the plasticized systems is significantly decreased (see Table 1).

### 3.2. Impedance Study

**Figure 2:**
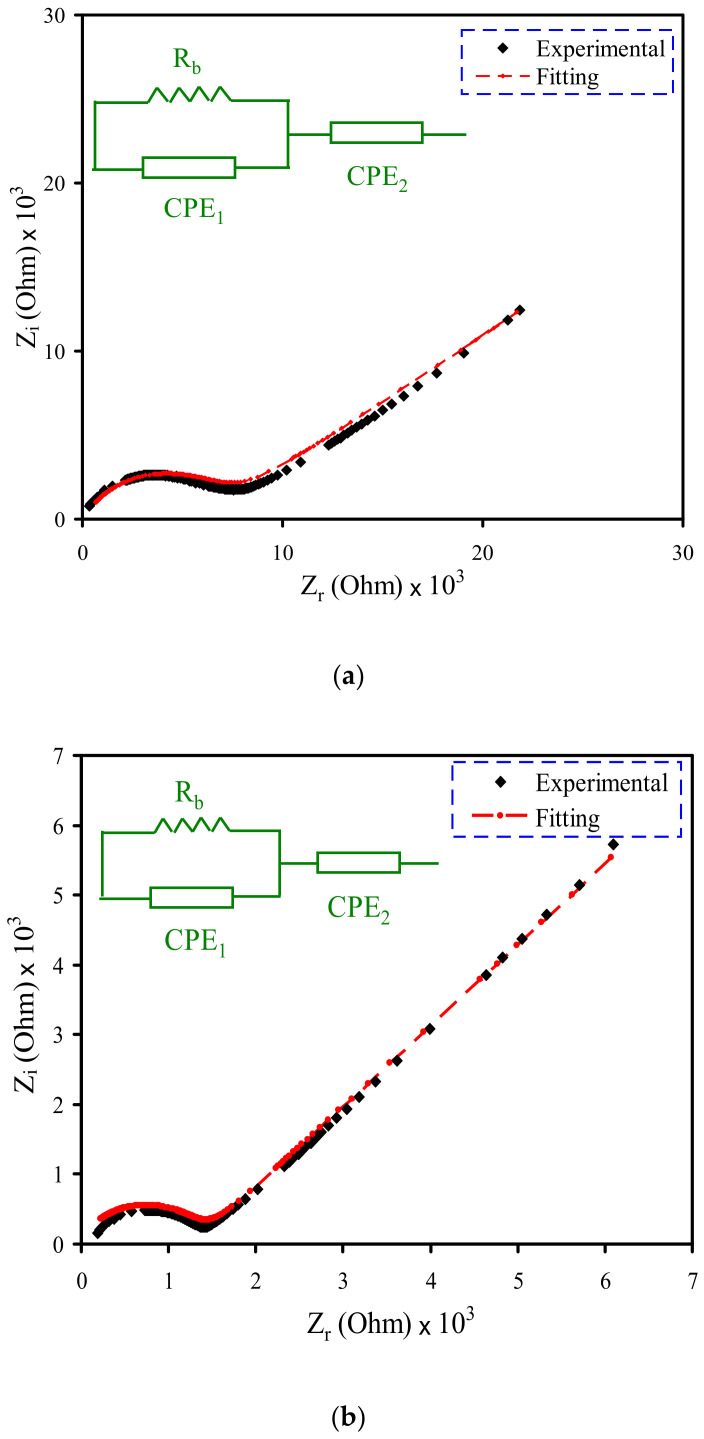

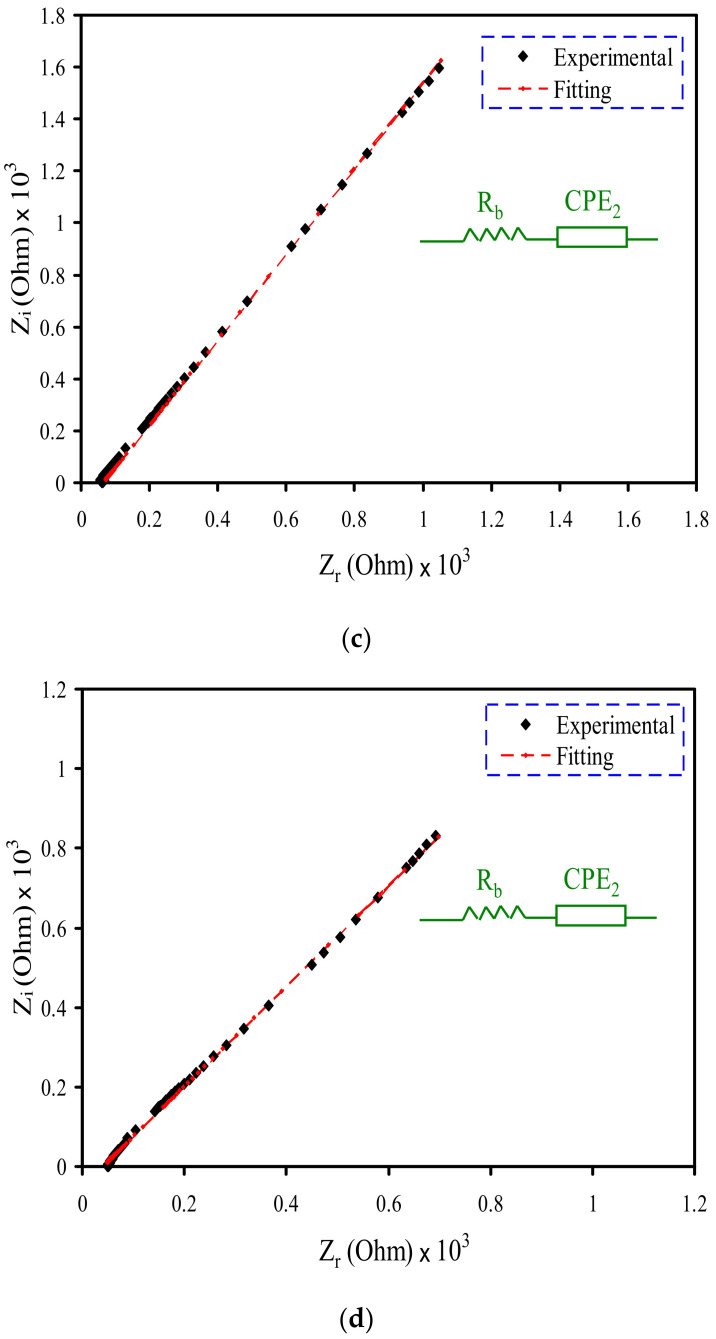
EIS plots for (**a**) PVSCGP0, (**b**) PVSCGP1, (**c**) PVSCGP2, and (**d**) PVSCGP3 electrolyte films.

During the investigation of electrolyte conductivity and the frequency behavior of the polymer electrolyte, impedance spectroscopy measurements were performed. The Cole–Cole plot (Nyquist plot) for each electrolyte system at room temperature is presented in Figure 2a–d. Considering the complex impedance plots, there is a high-frequency semi-circle and a low-frequency spike, as shown in Figure 2a,b. The semi-circle at the high-frequency is interrelated to a mixture of bulk resistance (due to migration of ions) and bulk capacitance (due to immobile polymer chains). While, at the low-frequency, a spike results from the effect of the charge polarization at the electrode–electrolyte interface [25,56]. When the 14 wt.% of glycerol is added to the electrolyte system, the diameter of the high-frequency semi-circle decreases (see Figure 2b). Furthermore, at 28 and 42 wt.% of glycerol, the semi-circle disappears, indicating the prevalence of the resistance within the polymer matrix, and the conductivity totally results from the ion mobility [57,58]. The values of bulk resistance (*R_b_*) are measured at the point where the semicircle intercepts the real axis (*Z_r_*). Equation (2) is employed to measure the films’ DC conductivity based on *R_b_* values and the dimensions of the film. Table 2 lists the DC conductivity for each film.

(2) $$\sigma_{dc}=\left(\frac{1}{R_{b}}\right)\times \left(\frac{t}{A}\right)$$

Concerning the low-frequency region, it is presumed that a straight line is parallel to the imaginary axis, i.e., the inclination of the straight line should be 90° in the complex impedance plots. To contrast, this inclination is caused by the double-layer capacitance (electrode polarization (EP) phenomena) at the blocking electrodes [59,60]. The fitting process of the impedance plots of the electrolyte systems taken from the experimental measurements is carried out in an attempt to obtain an electrical equivalent circuit (EEC) model. The equivalent circuit is used in the analysis because it is straightforward, providing a comprehensive profile of the systems [61]. The experimental impedance plots and the general equivalent circuit model of the SPE consist of a bulk resistance (*R_b_*) and two constant phase elements (CPE1 and CPE2), as shown in Figure 2a–d. Both *R_b_* and CPE1 responses are located in the high-frequency region. The response of CPE2 corresponds to the double-layer capacitance formed between the SPEs, and electrodes lie at the low-frequency spike region. Alternatively, the term constant phase element (CPE) is used in place of the capacitor in an equivalent circuit model. This is because in the SPE systems there are pseudo-capacitor or capacitor-like components rather than an ideal capacitor [62].

The impedance (*Z*) of CPE (*Z_CPE_*) is written as the following [63,64,65,66]:

(3) $$Z_{CPE}=\frac{1}{C\omega^{p}}\left[\cos\left(\frac{\pi p}{2}\right)-i\sin\left(\frac{\pi p}{2}\right)\right]$$

The values of real part (*Z_r_*) and imaginary part (*Z_i_*) are correlated to the equivalent circuit (inset of Figure 2a,b) that is mathematically expressed as below [53]:

(4) $$Z_{r}=\frac{R_{b}{}^{2}(A_{1})+R_{b}}{2R_{b}(A_{1})+A_{2}+1}+\frac{A_{3}}{C_{2}\omega^{p2}}$$

where

$$A_{1}=C_{1}\omega^{p1}\cos\left(\frac{\pi p_{1}}{2}\right),A_{2}=R_{b}{}^{2}C_{1}{}^{2}\omega^{2p1},\;\mathrm{and}\;A_{3}=\cos\left(\frac{\pi p_{2}}{2}\right)$$

(5) $$Z_{i}=\frac{R_{b}{}^{2}(A_{4})}{2R_{b}(A_{1})+A_{2}+1}+\frac{A_{5}}{C_{2}\omega^{p2}}$$

where

$$A_{4}=C_{1}\omega^{p1}\sin\left(\frac{\pi p_{1}}{2}\right)\;\mathrm{and}\;A_{5}=\sin\left(\frac{\pi p_{2}}{2}\right)$$

The spike features of the impedance of the plasticized electrolytes indicate that the resistive component of the polymer is predominant [25]. Based on this observation, the values of *Z_r_* and *Z_i_* in EEC (inset of Figure 2c,d) can be expressed as follows:

(6) $$Z_{r}=R+\frac{A_{3}}{C_{2}\omega^{p}}$$

(7) $$Z_{i}=\frac{A_{5}}{C_{2}\omega^{p}}$$

Considering Equations (3)–(7), the capacitance of the CPE is represented by *C*, ω is the angular frequency and *p* is interrelated to the plot deviation from the usual vertical axis in complex impedance plots. The EEC fitting parameters are shown in Table 3.

### 3.3. Dielectric Properties

To study the composition and physical and electrochemical properties of pure polymers and their corresponding blends, it is of great importance to evaluate the dielectric relaxation. It also is necessary and helpful to perform the dielectric relaxation and dipole relaxation measurements of polymer electrolytes over a wide frequency range [3,6,23].

**Figure 3:**
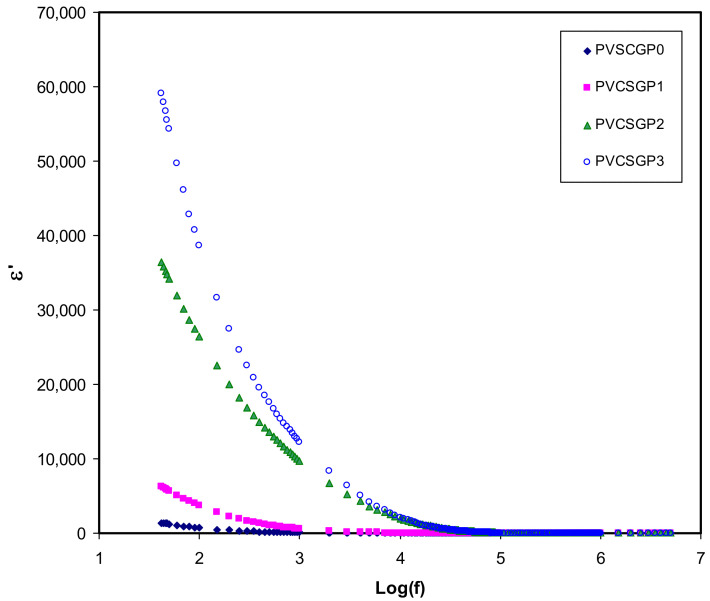
Dielectric constant versus log (f) for all polymer-blend electrolytes.

**Figure 4:**
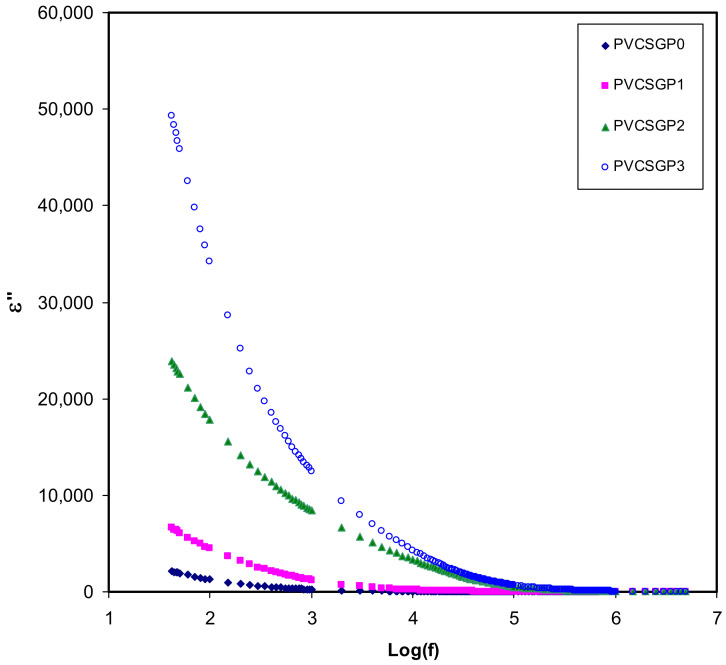
Dielectric loss versus log (f) for all polymer-blend electrolytes.

The dielectric spectroscopy is one of the useful and powerful techniques in dealing with the mechanism of ion transport [3,6,23,64]. To this aim, the complex dielectric constant (ε^*^) and complex electric modulus (M^*^) have to be measured. Figure 3 and Figure 4 show the dielectric constant (*ε*′) and dielectric loss (*ε*″) versus the frequency of electrolyte systems at room temperature. The values of *ε*′ and *ε*″ have been calculated using the equations shown below [3,6,23,67]:

(8) $$\varepsilon^{\prime }=\;\frac{Z^{\prime \prime }}{\omega C_{0}\;\left(Z^{\prime 2}+\;Z^{\prime \prime 2}\;\right)}$$

(9) $$\varepsilon^{\prime \prime }=\;\frac{Z^{\prime }}{\omega C_{0\;}\;\left(Z^{\prime 2}+\;Z^{\prime \prime 2}\;\right)}$$

where *ω* is the angular frequency, and *C_o_* is the vacuum capacitance (*C_o_* = *ε_o_A*/*d*, where *ε_o_* is a free space permittivity, 8.854187 × 10^−12^ F m^−1^). The value of *ε*’ and *ε*″ is relatively large within the low-frequency range; in contrast, as it is roughly a plateau at a higher frequency one. Interestingly, at low frequencies, charge accumulation from free mobile ions at the electrode–electrolyte interfacial region occurs as a result of electrode polarization, resulting in a thin layer of capacitance [68,69]. Contrarily, the reversal of the applied electric field is quick, and the majority of the ions may stay on in the bulk of the sample. This leads to lessening of the electrode polarization, decreasing the *ε*’ and *ε*″ value [3,6,23,70]. 

It is seen, as glycerol concentration is increased, the *ε*′ increases. This is owing to the increase in the number of charge carriers causing greater polarization [27,71,72,73]. Based on these results, it is easy to manipulate the conductivity of polymer electrolytes using the dielectric constant. It has already been proved that *ε*′ and the density of charge carriers (*n_i_*) are strongly associated with each other using a mathematical relationship,$n_{i}=n_{o}\exp(-U/\varepsilon^{\prime }K_{B}T)$, where U is the energy of dissociation [3,23,72,73]. Briefly, in other words, a decrease in *ε*′ results in a decrease in DC conductivity. The density of the charge carrier (*n_i_*) and their mobility (*µ_i_*) (*σ =* Σ *qn_i_µ_i_*), where q is the charge on the ion carriers, are two factors that govern the DC ionic conductivity of polymer ion-conducting electrolytes [3,23]. Thereby, a precise study of *ε*′ is useful in that one can achieve a complete understanding of the electrical properties of polymer electrolytes, particularly the conductivity.

**Figure 5:**
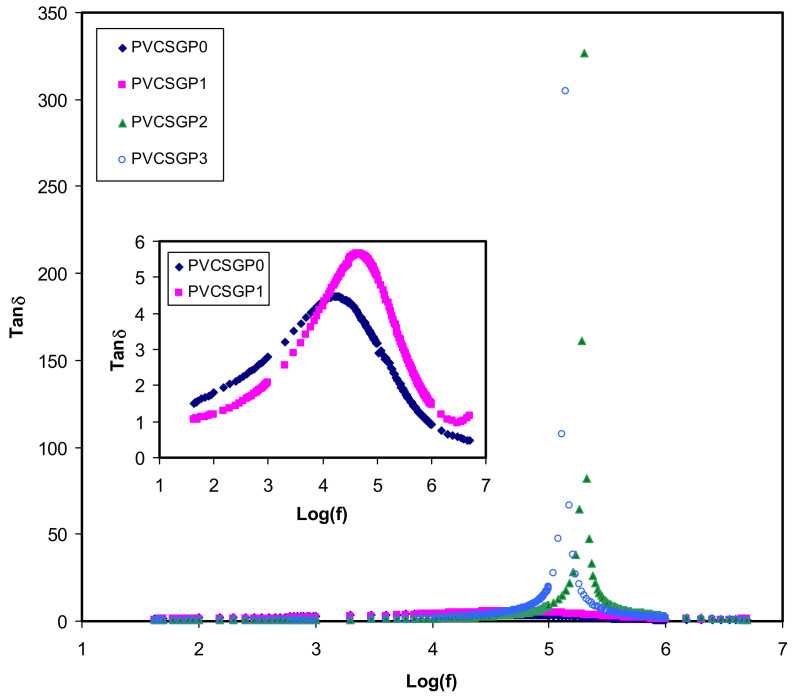
Tan δ versus log (f) for all polymer blend electrolytes.

The peaks of high electrically conductive polymer electrolyte systems are caused by either permanent or induced dipoles. These dipoles could be masked by the polarization relaxation of mobile charged species present in the material and, thus, the low-frequency relaxation peaks disappear [74,75]. Figure 5 shows the dielectric loss tangent (tan δ) versus frequency can be used to understand the relaxation process. Koop’s phenomenological model is used in the interpretation of the shape of tan δ [73,76]. According to the principles of the model, tan δ increases with increasing frequency until reaching a maximum value and then starts to decrease. It is explained based on the fact that at a low-frequency region, where tan δ increases, the ohmic component of the created current rises sharply compared to its capacitive component (*X_C_* = 1/2π*fC*). To contrast, at the high-frequency region, where tan δ decreases, the ohmic component is virtually frequency independent, and the capacitive component increases, resulting in a small value of *X_C_* [73,76,77]. Besides, the non-Debye type behavior of the relaxation process is evidenced by the broad nature of the tan δ peaks [41,78]. The complex impedance function is mathematically specified as Z^*^= *R* − j*X_C_*, where; *R* and *X_C_* are the resistor element and the capacitive element, respectively [73]. The above mathematical function of the impedance makes it clear that at low frequency the capacitive component is dominant and, thus, a major current passes through the resistor element. It is seen that from the tan δ = *ε*″/*ε*′ relationship, the tan δ is directly proportional to *ε*″.

Viewing the electric modulus, one can study the dielectric response that results from ion relaxation in which the electrode polarization effects can be suppressed; in other words, small features at the high-frequency region are recognized [79]. The impedance data correlate the real and imaginary parts of the electric modulus with each other through the equations shown below [10,47],

(10) $$M^{\prime }=\omega C_{0}Z^{\prime \prime }$$

(11) $$M^{\prime \prime }=\;\omega C_{0}Z^{\prime }$$

**Figure 6:**
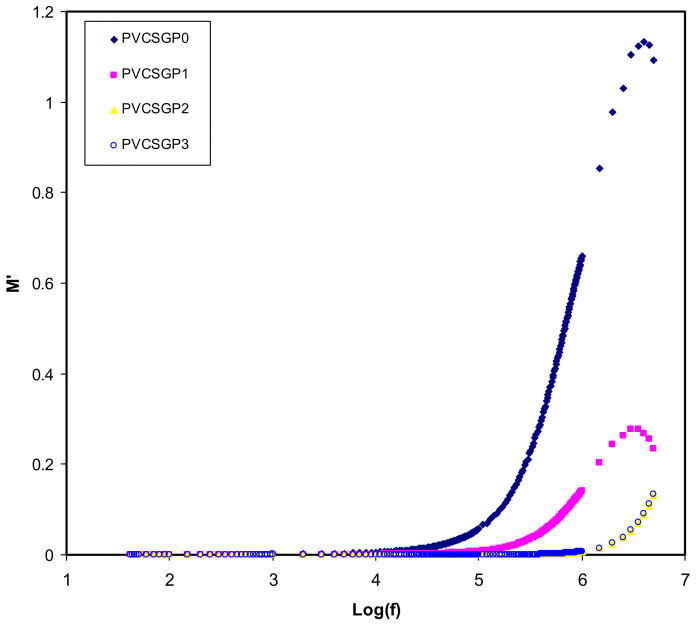
Real part of electric modulus versus log (f) for all polymer-blend electrolytes.

**Figure 7:**
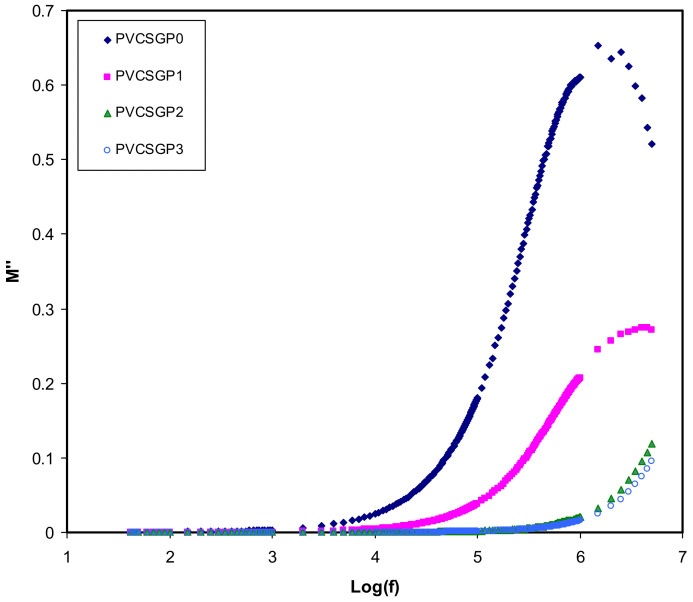
Imaginary part of electric modulus versus log (f) for all polymer-blend electrolytes.

Figure 6 and Figure 7 exhibit the frequency-dependence of real and imaginary parts corresponding to *M′* and *M″*, respectively, of the plot of the electrical modulus. The data points of the real part of the modulus spectra are located in the low-frequency region. This could be due to the large capacitance associated with the electrodes that facilitate the migration of the ion conduction process. It is seen that *M′* reaches a maximum saturation level in the high-frequency region. Occurring at the high-frequency region, the **ε*′* decreases to a minimum value and, thus, *M′* becomes maximum (*M_∞_* = 1/*ε_∞_*) [80], in other words. A dispersion in *M′* is observed when the frequency is increased, suggesting the non-Debye behavior of the samples [81]. The imaginary part of the modulus spectra is presented in Figure 7. It is seen that even at PVCSGP0 and PVCSGP1, the conductivity relaxation peaks appear. It also is notable that there is a shift of peak position to the higher frequency region with the addition of further glycerol concentration.

Fortunately, the electrical properties are supported by the XRD results. Presumably, in the *M″* spectra, some peaks would be concerning the translational ion dynamics and also reflect the conductivity relaxation of the mobile ions. During the amorphous phase, the segmental motion of the polymeric chain lowers the relaxation time and increases the ion transport process. To pinpoint, a mathematical relationship of τ = 1/2π*f*_max_, indicates that τ is the relaxation time of the ionic charge carriers [82]. Figure 7 shows the shift of the relaxation peaks to the lower frequency side. This is owing to the addition of glycerol that causes an increase in ionic conductivity that consequently reduces the relaxation time.

## 4. Conclusions

PVA-CS-NH_4_I glycerol-based plasticized electrolytes were successfully fabricated via the solution cast technique. The *X_C_* was observed to decrease with increasing plasticizer concentration. To attain more insight into the electrical properties of the films, the EIS spectra were fitted with electrical equivalent circuits (EECs). The EIS spectra analysis showed a decrease in bulk resistance, which reveals an increase of free ion carriers. The maximum conducting plasticized system showed a high conductivity of (1.37 × 10^−4^) S/cm, which is eligible for applications in energy storage devices, for example, supercapacitors and batteries. The outcome from the XRD examination indicated that the maximum conducting plasticized system exhibited the minimum *X_C_,* which was determined to be 1.34. The XRD outcomes also can be associated to the trend in the *X_C_* with a variation in the conductivity of the electrolyte. The conductivity trend was further confirmed via dielectric examination. Concerning the region of low frequencies, large values of dielectric loss and dielectric constant were obtained owing to electrode polarization. Regarding the electric modulus and loss tangent plots, the distribution of relaxation times associated with conducting ions were concluded.

## Figures and Tables

**Table 1:** The degree of crystallinity is calculated from the deconvoluted XRD pattern.

Electrolyte	Degree of Crystallinity (%)
Pure PVA	41.68
Pure CS	15.97
PVA:CS	15
PVSCGP0	14.39
PVSCGP1	11.01
PVSCGP2	7.37
PVSCGP3	1.34

**Table 2:** DC conductivity for the electrolyte systems at room temperature.

Designation	Conductivity (S cm^−1^)
PVSCGP0	9.75 × 10^−7^
PVSCGP1	5.02 × 10^−6^
PVSCGP2	7.81 × 10^−5^
PVSCGP3	1.37 × 10^−4^

**Table 3:** The EEC fitting parameters for electrolyte systems at room temperature.

Sample	*K1 (F* ^−1^ *)*	*K2 (F* ^−1^ *)*	*C1 (F)*	*C2 (F)*
PVSCGP0	2 × 10^8^	2.3 × 10^5^	5 × 10^−9^	4.3 × 10^−6^
PVSCGP1	1.98 × 10^8^	1.7 × 10^5^	5.05 × 10^−9^	5.88 × 10^−6^
PVSCGP2		8.1 × 10^4^		1.23 × 10^−5^
PVSCGP3		2.85 × 10^4^		3.51 × 10^−5^
